# *SCN2A* channelopathies in the autism spectrum of neuropsychiatric disorders: a role for pluripotent stem cells?

**DOI:** 10.1186/s13229-020-00330-9

**Published:** 2020-04-07

**Authors:** Karina A. Kruth, Tierney M. Grisolano, Christopher A. Ahern, Aislinn J. Williams

**Affiliations:** 1 Department of Psychiatry, Iowa Neuroscience Institute, University of Iowa, 169 Newton Rd, 2326 PBDB, Iowa City, IA 52242 USA; 2 Department of Molecular Physiology and Biophysics, Iowa Neuroscience Institute, University of Iowa, 169 Newton Rd, 2312 PBDB, Iowa City, IA 52242 USA

**Keywords:** *SCN2A* syndrome, Autism spectrum disorder, Induced pluripotent stem cell, Sodium channel, *SCN2A*, Na_V_1.2, Organoid, Cell model

## Abstract

Efforts to identify the causes of autism spectrum disorders have highlighted the importance of both genetics and environment, but the lack of human models for many of these disorders limits researchers’ attempts to understand the mechanisms of disease and to develop new treatments. Induced pluripotent stem cells offer the opportunity to study specific genetic and environmental risk factors, but the heterogeneity of donor genetics may obscure important findings. Diseases associated with unusually high rates of autism, such as *SCN2A* syndromes, provide an opportunity to study specific mutations with high effect sizes in a human genetic context and may reveal biological insights applicable to more common forms of autism. Loss-of-function mutations in the *SCN2A* gene, which encodes the voltage-gated sodium channel Na_V_1.2, are associated with autism rates up to 50%. Here, we review the findings from experimental models of *SCN2A* syndromes, including mouse and human cell studies, highlighting the potential role for patient-derived induced pluripotent stem cell technology to identify the molecular and cellular substrates of autism.

## Background

Our understanding of autism spectrum disorders (ASD) has increased exponentially over the last 20 years. We now know that though a clear diagnosis may not be made until age two or later, developmental dysfunction may begin far earlier [1]. Researchers have identified hundreds of risk genes for autism, and increasing evidence indicates that parental age, prenatal exposure to certain medications, and even pollution also affect the likelihood an individual will develop the disorder [2–6]. Only a small proportion of autism cases are clearly linked to a single genetic mutation; the vast majority of cases are complicated, likely the result of a combination of multiple genetic and environmental factors [7].

The complex etiology of autism presents a significant challenge to researchers and clinicians alike: there are hundreds of possible relevant variables to control for and consider, while pharmaceutical treatments are difficult to develop in the absence of genetic or molecular target(s). Recent research suggests that neurodevelopmental abnormalities associated with ASD begin in utero [1]. Furthermore, earlier treatment is correlated with greater independence later in life [8], suggesting that some aspects of dysfunction can be ameliorated by appropriate treatment during key windows or sensitive periods in brain development. It is thus increasingly apparent that autism is a disorder of early brain development, and as such, our scientific efforts need to likewise be directed toward development. While a comprehensive review of ASD is beyond the scope of this review, we refer readers to several excellent recent reviews for further detail on ASD genetics, etiology, and phenotype [2, 8–10].

Animal models have yielded great strides toward our understanding of ASD, both mechanistically and behaviorally. However, as we have learned from decades of translational research, drugs that appear promising in animal models often fail in clinical trials due to adverse effects or lack of efficacy in humans. Human subject studies, and in particular functional MRI (fMRI) experiments, have shed light on the differences between brain function in individuals with typical cognition versus those with autism. However, human studies are limited by a variety of factors, including the ethics and challenges of working with small children or infants, high subject-to-subject variability, and the inability to obtain live primary tissue for in vitro experiments.

Human embryonic stem cell (hESC) research first opened the door to in vitro experiments with human neurons as researchers developed techniques to differentiate hESCs into neurons. In 2007, the ability to transform differentiated human cells such as fibroblasts into induced pluripotent stem cells (iPSCs) changed the playing field [11–13]. For the first time, scientists were able to generate different cell types from an individual who had (or had not) been diagnosed with a particular condition of interest. Stem cells produced from ASD subjects already contain at least one of the genetic cocktails needed to generate autism phenotypes, and thus the cell types produced through subsequent differentiation of these stem cells should likewise be more representative of neurons from an individual with ASD and therefore may facilitate the study of autism on a molecular and cellular level.

Despite the advances provided by iPSCs, significant caveats remain. For one, multiple iPSC clones derived from the same subject show significant clone-to-clone variability [14–16]. Additionally, as with fMRI and other human subject studies, there is variability between study subjects. A further complication is found in the variety of factors which contribute to the development of autism: subjects diagnosed with ASD likely converge on a similar phenotype through different paths. Thus, even with the use of human-derived iPSC lines, high variability between subjects and samples might generate enough experimental “noise” that critical phenotypes may be missed. To maximize the effectiveness of iPSCs for studying autism-linked genes, it may be necessary to focus on less genetically complex forms of ASD to reduce variability.

Although it is difficult to minimize variability between iPSC clones, a meaningful step toward enhancing experimental signal can be achieved by narrowing the scope of the disorder in question from the very broad umbrella diagnosis of ASD to a single model with a simple, clearly identified etiology. A small subset of autism cases are monogenic in origin and can be directly tied to a single causal mutation [5], such as loss-of-function mutations in *SCN2A* which cause *SCN2A* syndromes [17–19]. While such cases are often on the severe end of the phenotypic spectrum, the classical characteristics of autism such as repetitive behaviors and interests, difficulty with social cues, and rarely initiating social interaction, are present and consistent with more common forms of ASD. Stem cell models of monogenic forms of ASD may allow researchers, in combination with careful isogenic controls, to subtract out a significant amount of experimental noise caused by genetic and environmental variability. Monogenic ASD iPSC lines can then be used for making isogenic comparison lines using CRISPR or TALENs, which allow the experimenter to control for genetic background. While this does not eliminate all variability, it does allow stronger inferences to be made about the specific effect of a mutation in a single gene, especially in situations where there are multiple donors from which multiple isogenic lines can be created.

The eventual long-term goal of using iPSC models for *SCN2A*-related diseases is to expand the technique from modeling monogenic diseases to modeling more complex polygenic diseases. However, as highlighted by Zhu et al. in their thoughtful review of the subject [20], cell culture phenotypes are often much more subtle than clinical phenotypes, which can make them difficult to detect and quantify in vitro. In this case, the use of monogenic models is helpful, and potentially even critical, as a first step toward developing in vitro assays which detect cellular and molecular phenotypes that can be correlated with clinical phenotypes. Once this initial foundation has been laid, more variables can be introduced to the experimental system, such as disease models with multiple genetic or environmental factors. As Zhu et al. suggest, the original monogenic models can then serve as a form of positive control against which more complex models can be compared. iPSC models have already been successfully used to identify cellular and molecular dysfunction that occurs in other forms of monogenic ASD-related disorders, including fragile X syndrome [21–24], Timothy syndrome [25–28], Rett syndrome [29–32], tuberous sclerosis [33–36], and neurofibromatosis type 1 [37–40]. As more monogenic models are added to the list, in vitro phenotypes can be compared and contrasted between the models to better understand the phenotypes from autism models with a more complex etiology.

To date, the Simons Foundation for Autism Research has listed 174 genes as “high confidence” for association with autism spectrum disorders [41]. *SCN2A*, which encodes the voltage-gated sodium channel Na_V_1.2, is of particular interest as an in vitro model for several reasons, including the following: (1) It is among the most common monogenic sources of autism-causing mutations [19]; (2) Gain-of-function and loss-of-function mutations have both been identified in humans but with markedly different phenotypes (epilepsy vs autism/intellectual disability, respectively) [17], allowing for a compare-and-contrast opportunity between variants; (3) Several mouse models already exist which may complement iPSC models [42–46]; (4) Sodium channels are “druggable” targets [47–50]; and (5) Elucidation of the role of *SCN2A* in neural development and function may also contribute to the development of therapies to treat *SCN2A*-related diseases such as epilepsy, which is often intractable [51].

### *SCN2A* and Na_V_1.2

**Fig. 1:**
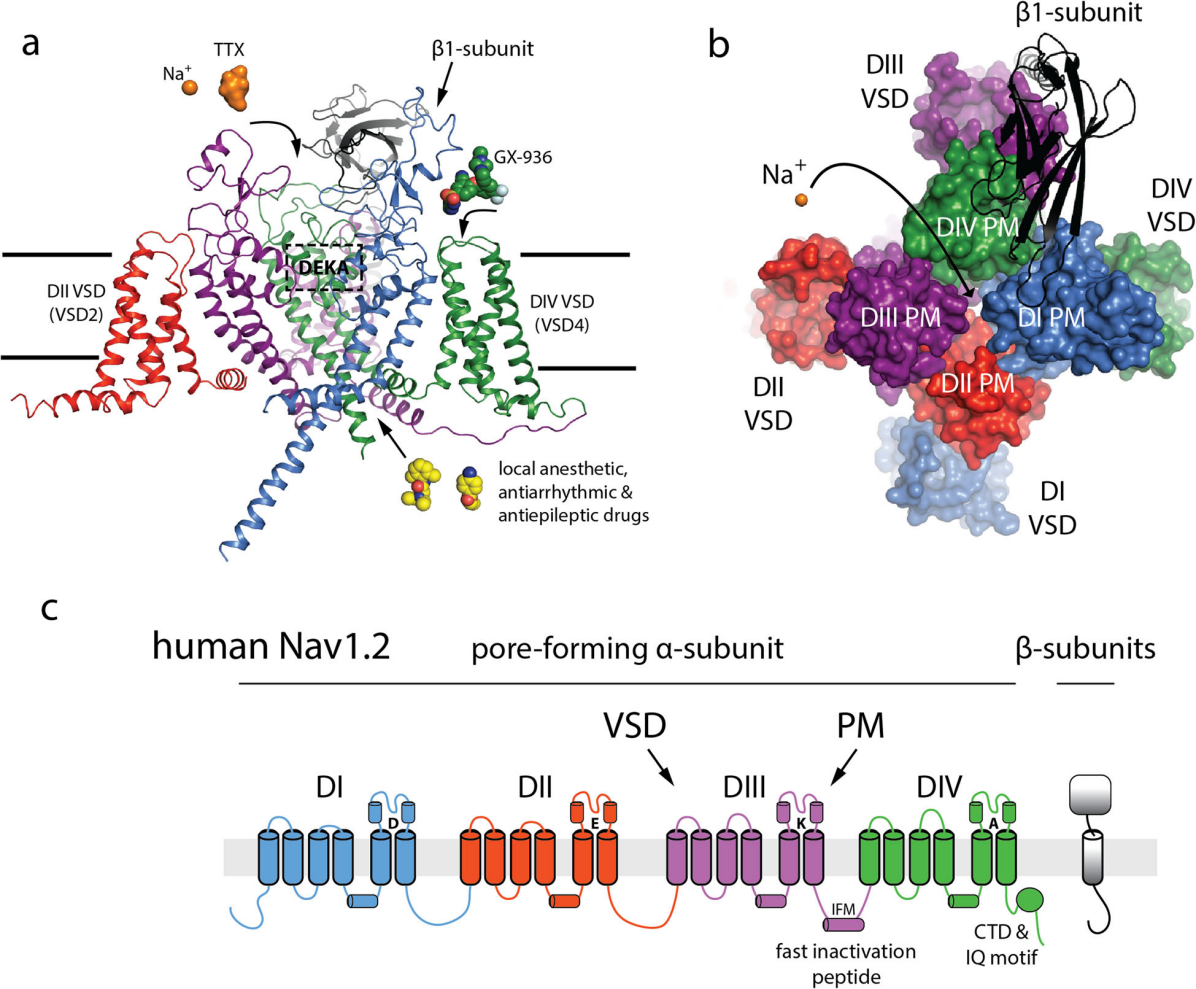
Structure, architectural elements, and sodium channel (Na_V_) topology. **a** Side view of the voltage-gated sodium channel pore forming (alpha) subunit. Each domain is color depicted. Extracellular IG-fold domain of the beta subunit is shown. A sodium ion and tetrodotoxin (TTX), both in orange, are shown outside of the external vestibule. GX-936, a potent and isoform-selective acylsulfonamide, is shown near its DIV voltage-sensor domain (VSD) binding site. The DEKA box represents the selectivity filter comprised of Asp, Glu, Lys, and Ala sidechains. Traditional sodium channel blockers are shown at the intracellular entrance to the of pore domain. **b** Top view of (**a**) depicting the four-fold symmetry and central permeation pathway for by the Pore Module (PM) of each domain and voltage-sensing domain (VSD). Note the domain swapped architecture between each PD and VSD, a feature which underlies allosteric coupling in Na_V_ gating. **c** Classic topology plot showing the same features

*SCN2A* encodes the voltage-gated sodium channel Na_V_1.2, which contains 27 exons encoded by 2005 amino acids [Fig. 1]. Sodium channels are large (~ 250 kD) membrane proteins composed of 24 transmembrane (TM) segments that are organized into four homologous domains (DI-DIV). Each domain contains 6 TM segments where the first four TM segments (S1–S4) make a voltage sensor, and the second two segments (S5 and S6) create a central ion pore. The pore domain houses the “selectivity filter,” an unusual protein fold which helps to select for sodium over other monovalent cations, as well as the “gates” formed by the S6 bundle-crossing which control the transmembrane flow of sodium ions. The status of the central gate is tightly coupled to voltage-sensor domains which display sub-millisecond activation in response to depolarizing stimulus. The resulting inward flow of cationic sodium ions down their electro-chemical gradient into the neuron—roughly one million ions per second for each channel—produces a rapid depolarization of the membrane, seen as the upstroke of the action potential. After opening (within milliseconds), sodium channels spontaneously inactivate (cease to conduct) to curb the inward flow of more sodium ions and to allow for membrane repolarization. The overall gating scheme and protein architecture is conserved for all nine mammalian sodium channel isoforms [52].

Na_V_1.2 is predominantly expressed in the brain, specifically in cortical pyramidal neurons and cerebellar granule neurons [53–56]. There are two known developmentally relevant splice isoforms, “neonatal” (5N) and “adult” (5A), distinguished by which of two mutually exclusive variants of exon 5 they contain. As can be inferred by their names, the neonatal variant is the dominant isoform found in cortical neurons during early development, and this is gradually exchanged for the adult variant after birth [57]. In contrast, in cerebellum, the adult isoform is approximately twice as abundant as the neonatal isoform at birth, and the proportion of 5A only slightly increases during development [57]. Studies have shown that the 5N variant, which contains an asparagine (N) at residue 209, is less excitable than its adult counterpart, which contains an aspartic acid (D) at residue 209 [44]. Mice engineered to express the 5A variant throughout development display an increased likelihood of developing seizures that does not abate after development, suggesting permanent developmental effects occur as a result of the specific temporal expression patterns of the two isoforms [44].

During early development, Na_V_1.2 is localized along the axon length in cortical pyramidal neurons [58]. As development continues, Na_V_1.2 is gradually replaced by Na_V_1.6 along the axon, and Na_V_1.2 expression becomes localized mainly to the axon initial segment [59, 60]. Recent work by Spratt et al. suggests that Na_V_1.2 enhances backpropagation of action potentials in cortical pyramidal neurons, and thus Na_V_1.2 may play a role in synaptic plasticity and learning [61]. In contrast, in cerebellum, Na_V_1.2 expression persists at a much higher level in adulthood [57]. Combined with the different expression patterns of the 5N/5A isoforms in cerebellum, these data suggest the channel may play different roles in the different regions of the brain.

The complex nature of Na_V_1.2 expression, both in the ratio of 5N/5A isoforms and its replacement with Na_V_1.6 in certain brain regions, suggests there is a fine-tuned balance of these channels during brain development, and any disturbance of this regulation might result in some form of developmental defect. It is little wonder, then, that mutations in *SCN2A* lead to intellectual disability, epilepsy, and autism.

### *SCN2A* syndrome(s)

In general, mutations that alter neuronal sodium channel structure, function, or expression lead to epilepsy and neurological disorders, but most *SCN2A* mutations that cause autism result from mutations that produce premature stop codons (a.k.a. nonsense mutations), which cause termination of protein translation. This effectively results in Na_V_1.2 haploinsufficiency (homozygous loss of Na_V_1.2 appears incompatible with life) [45]. As of 2018, 276 patients were identified with *SCN2A*-mediated disorders, approximately 50% of whom displayed autism [19]. However, the current rates of *SCN2A* mutations in both ASD and other neurodevelopmental disorder populations are likely underestimated, and the incidence is expected to increase significantly as genetic testing for these patients becomes more widespread. Indeed, Sanders et al. estimate that disease-causing *SCN2A* mutations may occur as frequently as 7.5 per 100,000 births in the general population [19]. Of the 50% of *SCN2A* syndrome patients diagnosed with ASD, their symptoms are similar to those seen in more common forms of ASD, such as reduced social interaction and repetitive behaviors. Some patients also exhibit additional symptoms such as intellectual disability (ID) and developmental delay, seizures, and symptoms of cerebellar dysfunction such as abnormal gait, clumsiness, and hypotonia [19].

Of the *SCN2A* mutations linked to autism, intellectual disability, and/or epilepsy, many of these variants appear to be de novo, rather than inherited. In 2014, Baasch et al. performed exome sequencing of a patient with multiple symptoms including seizures, severe intellectual disability, structural abnormalities in the brain, as well as abnormal brain activity via EEG [62]. They discovered a de novo missense mutation in *SCN2A* that had not been previously reported. Based on their conclusions and literature review, they suggest that de novo mutations of *SCN2A* are linked to more severe phenotypes. Likewise, Nakamura et al. also suggest that inherited *SCN2A* mutations result in less severe phenotypes than de novo *SCN2A* mutations [63]. This also suggests that apparent rates of *SCN2A* mutations may increase as new deleterious variants are identified.

It has been proposed that within *SCN2A* mutations, those that cause a gain of function and increased neural excitability cause epilepsy, whereas mutations that cause loss of function, either dampening channel function or leading to haploinsufficiency, cause autism [17]. However, Wolff et al. identified several loss-of-function mutations that cause later-onset epilepsy, suggesting that the relationship between channel function and phenotype may be more complicated [51]. Emerging evidence suggests that *SCN2A* may also contribute to schizophrenia and intellectual disability in the absence of autism [64, 65], although these associations require further investigation [66]. Nevertheless, the variety of phenotypes associated with different types of *SCN2A* mutations may provide an opportunity to more fully investigate the relationship between channel function and phenotype.

### *SCN2A* studies in humans

Histological postmortem examination of Na_V_1.2 in hippocampus and temporal lobe shows that expression begins in these brain regions at 19 weeks of gestation, peaks at 7–9 months of age, is sustained throughout childhood, and gradually decreases in adulthood [56]. Although EEG has been used in *SCN2A* epilepsy patients to characterize neural dysfunction during seizures [63, 67], it has not been used to measure brain activity in nonepileptic states. MRI studies have revealed that many *SCN2A* syndrome patients display structural abnormalities in the brain, including (from most to least common) cerebral atrophy, thin corpus callosum, delayed myelination, and cerebellar atrophy [63].

In one study assessing the effects of different *SCN2A* variants in schizophrenia, postmortem examination of *SCN2A* expression in the prefrontal cortex revealed that lower levels of *SCN2A* expression were associated with poorer cognitive performance [64]. Subsequent fMRI studies showed that the *SCN2A* SNP rs10174400 is a significant predictor of cognitive ability not only in schizophrenia patients but in healthy adults as well [64, 68]. Combined, these results suggest that common variation in *SCN2A* plays a role in cognitive ability, which may explain why mutations in *SCN2A* often cause intellectual disability.

Although it is true that many of the characteristics of *SCN2A* patients are not representative of more common (and less severe) forms of autism, the distinctive nature of the phenotypes observed with *SCN2A* syndrome facilitates our ability to connect phenotypes observed in experimental models to those observed clinically in patients. These distinct phenotypes can then be compared and contrasted with those observed in other monogenic ASD models (and eventually, to complex polygenic models) to determine which characteristics are unique to *SCN2A* syndrome and which are more broadly relevant to ASD in general. As more monogenic ASD models are compared and contrasted, it will be easier to identify those characteristics common to autism specifically versus those belonging to each unique syndrome. The broader developmental dysfunction phenotypes observed in *SCN2A* syndromes may also serve as a model to aid our understanding of developmental dysfunction in other neurodevelopmental disorders with which it is genetically linked, such as intellectual disability and schizophrenia.

### Mouse models of *SCN2A* syndrome

#### Scn2a^Q54^

The longest standing mouse model used to investigate the role of *SCN2A* in vivo is the *Scn2a*^*Q54*^ mouse, which expresses a gain-of-function *SCN2A* mutant*,* GAL879-881QQQ, at a low level over a WT *Scn2a*^*+/+*^ background. *Scn2a*^*Q54*^ mice exhibit epilepsy, persistent sodium current, spontaneous action potential firing, and repetitive behaviors [69]. To date, these mice have primarily served as a model for epilepsy, and there are no published studies investigating ASD endophenotypes in *Scn2a*^*Q54*^ mice. Multiple genetic modifiers that influence the severity of *Scn2a*^*Q54*^ epilepsy have since been discovered, including Kcnq2 [70], Cacna1g [71], and Hlf [72]. Interestingly, the severity of the *Scn2a*^*Q54*^ phenotypes produced are highly dependent upon mouse strain: *Scn2a*^*Q54*^ mice on the SJL/J background exhibit more frequent and more severe seizures than *Scn2a*^*Q54*^ mice on a C57BL/6J background [73, 74]. Further investigation into the differences between the two models has identified calcium/calmodulin protein kinase II (CaMKII) as a modulator of the *Scn2a*^*Q54*^ phenotype [74], suggesting the CaMKII pathway may serve as a therapeutic target for *SCN2A*-associated epilepsy.

### Haploinsufficient mouse models

Homozygous knockout of *SCN2A* is perinatally lethal [45], prohibiting the use of complete, unconditional gene knockout for all but in vitro assays. Nevertheless, *Scn2a* haploinsufficient mouse models (*Scn2a*^*+/−*^) show significant phenotypes linked to human disease and have been used to further elucidate the role of Na_V_1.2 in both normal neurodevelopment and disease. *Scn2a*^*+/−*^ mice display mild absence-like seizures, delayed spatial learning, increased contextual fear learning, impaired fear extinction, and hyperactivity [42, 46, 75, 76], all of which may be related to ASD symptoms in humans. Cortical neurons from these mice display impaired excitability and impaired excitatory synapse function [61]. Additionally, *Scn2a*^*+/−*^ mice display reduced anxiety in novel environments [42, 43] but show an increased aversion to light [42], suggesting the model may recapitulate some of the sensory sensitivity seen in ASD.

Other potential endophenotypes of ASD, such as reduced sociability, impaired communication, and repetitive behaviors, are not consistently observed in *Scn2a*^*+/−*^ mice. Léna and Mantegazza found that *Scn2a*^*+/−*^ mice displayed repetitive behaviors and reduced vocalization and communication as juveniles, but that these phenotypes disappeared in adulthood [43]. In contrast, Tatsukawa et al. did not observe repetitive behaviors or communication deficits in juvenile or adult *Scn2a*^*+/−*^ mice [42]. Therefore, it is unclear whether simple loss of one copy of *SCN2A* is sufficient to generate the social and behavioral phenotypes of autism spectrum disorders. More work is needed, possibly with specific genetic models of ASD-linked *SCN2A* mutations, to fully address specific differences in genotype-phenotype relationships.

### Non-mammalian *SCN2A* models

An *SCN2A* homolog occurs in both zebrafish (*scn1Lab*) and *Drosophila* (*paralytic*). In both organisms, the homolog is not specific to *SCN2A* but instead bears significant homology to three human sodium channel counterparts: *SCN1A*, *SCN2A*, and *SCN3A*. Knockout models have been created for both organisms (reviewed in [77]), but to date, these have generally been used as models for *SCN1A* but not *SCN2A* or *SCN3A*.

Mutations in the *SCN1A* gene, which encodes the voltage-gated sodium channel Na_V_1.1, lead to Dravet syndrome, which is characterized by temperature-sensitive epilepsy. Both zebrafish and *Drosophila* models for Dravet syndrome display seizure phenotypes which respond to drugs used to treat Dravet epilepsy, suggesting the models may be effective for drug screening [78].

Interestingly, based on MUSCLE homology analysis [79], zebrafish *scn1Lab* is slightly more homologous to human *SCN2A* than *SCN1A*, with 81% and 77% identities, respectively. The high sequence homology to *SCN2A* suggests that these zebrafish models may be useful tools for drug screening in the context of *SCN2A*-related disorders, as well. The *Drosophila paralytic* gene is much less homologous to human sodium channels, with 57% identity to *SCN2A* and 47.5% identity to *SCN1A*. However, Sun et al. [80] and Schutte et al. [81] were able to recapitulate temperature-sensitive seizure phenotypes in *Drosophila* using knock-in mutations homologous to Dravet-causing mutations in human *SCN1A*. These models were subsequently used to further elucidate neural dysfunction in Dravet syndrome. In summary, while zebrafish and *Drosophila* may not serve as an ideal behavioral model for *SCN2A*-related disorders, they may prove useful for drug screening and as a tool to identify genetic interactions.

### Cell-based *SCN2A* models

#### Human stem cell models of SCN2A mutations

To date, one patient-derived *SCN2A* syndrome iPSC line has been published, though no data have yet been published using it as an experimental model [82]. However, a number of investigators have used CRISPR to genetically modify the *SCN2A* gene in stem cells derived from control subjects, which have revealed phenotypes that mirror those observed in mouse models of *SCN2A* syndrome mentioned above. In 2018, Deneault et al. used CRISPR to create a homozygous knockout of *SCN2A* in a human iPSC line generated from the unaffected father of a child with ASD [83]. These stem cells were subsequently differentiated into excitatory neurons through ectopic expression of *NEUROG2* and assessed for neural activity. The authors found that, compared to neurons generated from the control *SCN2A*^+/+^ line, *SCN2A*^−/−^ neurons showed a significant reduction in spontaneous excitatory postsynaptic current, mean firing rate, and network burst frequency. Based on both patch-clamp and multi-electrode array analyses, Deneault et al. concluded that knockout of *SCN2A* led to an overall decrease in neuronal activity at both the single cell and population levels [83].

In 2019, Lu et al. used CRISPR to create an *SCN2A* haploinsufficient (*SCN2A*^+/−^) hESC line [84]. Using overexpression of *NEUROG1* and *NEUROG2*, the authors differentiated the cell line into a network of both glutamatergic excitatory and GABAergic inhibitory neurons. Similarly to what Deneault et al. reported with *SCN2A*^−/−^ neurons [83], Lu et al. found that *SCN2A*^+/−^ neurons also displayed a significant reduction in spontaneous neural activity. However, *SCN2A* haploinsufficiency did not appear to disrupt inhibitory synaptic transmission in GABAergic cells. Lu et al. thus concluded that haploinsufficiency of *SCN2A* is sufficient to cause an overall decrease in neural network activity.

These two studies demonstrate that both hESCs and iPSCs can be used to create in vitro models to study *SCN2A* activity in neurons on a human genetic background. Furthermore, the consistency in results between both studies—from two different groups using completely different cell lines—supports the use of induced neurons from stem cells as a viable model system. What is unknown from these models is how disease-causing mutations from actual *SCN2A* syndrome patients affect the channel’s electrophysiological properties, and whether the mutations observed in patients are associated with disruptions of other intracellular signaling pathways or channel functions.

### The future of *SCN2A* iPSCs

While stem cell models are already proving valuable, the ability to direct differentiation into specific, desired cell types is still relatively new. Many types of neurons have yet to be reliably produced in vitro, and existing differentiation protocols often yield heterogeneous populations of neurons. As proper neuronal development and function often depends on the interplay between different cell types, future efforts should focus on creating more physiologically relevant in vitro neural environments. Specifically, since Na_V_1.2 is primarily expressed in glutamatergic neurons of the cerebral and cerebellar cortex, and these neurons develop in tandem with GABAergic neurons and glia, it would be ideal to utilize model systems in which these neurons differentiate together in the ratios normally observed in the embryonic brain, and if possible, in their normal three-dimensional physical relationships.

In addition to the difficulty in generating specific cell types, in vitro neural differentiation often results in functionally immature neurons. Generally speaking, iPSC-derived neurons have depolarized resting membrane potentials, high input resistance, low membrane capacitance, and are often unable to fire repetitive, narrow-spike-width action potentials [85–90], all of which suggest that they may not express the full complement of ion channels found in mature neurons despite having typical neuronal morphology. For this reason, induced neurons may be a good tool to investigate neurodevelopment in vitro, but they are currently a poor model for mature neural networks. It may be that physiologically relevant heterogeneous populations of neurons are required to achieve neural maturity from stem cells. It is also probable that three-dimensional structure affects development, and thus more elaborate and multi-dimensional culture methods will likely need to be utilized.

### Organoids

As discussed above, neurodevelopment occurs in a tightly temporally and spatially regulated manner, and aberrant Na_V_1.2 expression (either high or low) is likely to disrupt this process in various brain regions. While this process can be studied in non-human animal models, these do not recapitulate the human genetic context that likely contributes to *SCN2A* syndrome. To study the role of Na_V_1.2 in human neurodevelopment, organoid models seem particularly promising. Organoids are three-dimensional systems in which iPSCs partially regulate their own differentiation into a specific tissue type, resulting in a model tissue that closely recapitulates the normal process of development. The majority of brain organoid protocols generate tissues with gene expression patterns most similar to the second trimester of in utero development or younger [91–94]. Recent technical improvements in organoid technology and prolonged periods in culture may enable scientists to model later stages of fetal development [95]. Organoids have been used to model both idiopathic autism [96] and autism related to mutations in the voltage-gated calcium channel Ca_V_1.2, a.k.a. Timothy syndrome [25], and studies suggest that these models will be useful for studying both neurodevelopmental and genetic factors that contribute to ASD [97]. Indeed, cortical organoids that were developed using iPSCs derived from patients with CDK5RAP2-associated microcephaly accurately recapitulated human phenotypes that were not apparent in animal models likely due to differences in neurodevelopment, highlighting the relevance of organoid technology [98]. In the case of *SCN2A* syndrome, organoids may help reveal both cell-autonomous and non-cell-autonomous effects of Na_V_1.2 function. For example, although Na_V_1.2 is mostly expressed in glutamatergic neurons, we do not know whether loss of Na_V_1.2 in glutamatergic neurons might have downstream effects on the development or function of GABAergic neurons or glia, which tend to develop later. In the context of organoids, it is possible to detect how abnormalities in one cell type affect another cell type in a human developmental context.

### Neural Differentiation

In addition to organoids, iPSCs can be differentiated in 2D cultures, allowing for experiments that may be challenging in 3D organoids, such as patch-clamping of individual neurons. Although neurons can be isolated and cultured from animal models, human iPSC-derived neurons have been shown to successfully recapitulate human phenotypes that did not manifest in the animal model, such as with 16p11.2del models [99]. The available data suggest that the gene product of *SCN2A*, Na_V_1.2, is primarily expressed in glutamatergic neurons of the cerebral and cerebellar cortex. Fortunately, forebrain cortical glutamatergic neurons are one cell type for which the field has developed robust and reproducible iPSC differentiation protocols. Overexpression of *NEUROG2* can drive consistent and rapid maturation of this neuronal type [100], which may help reveal how Na_V_1.2 is localized and functions in human neurons. It is important to note, however, that *NEUROG2* overexpression protocols may bypass developmental stages of interest and thus are likely best suited to studies of ion channel localization and function rather than neurodevelopment. The role of Na_V_1.2 during early neurodevelopment may be better recapitulated using differentiation protocols that rely on small molecules and morphogens, such as dual-SMAD inhibition and dorsalization via small molecules [101, 102].

The other brain region noted for persistently high levels of Na_V_1.2 expression is the cerebellar cortex, specifically, cerebellar granule neurons. As described above, these cells express Na_V_1.2 throughout development and maintain high expression of this channel throughout life, rather than replacing it with Na_V_1.6 (as occurs in the cerebral cortex). There are a small number of published protocols for generating cerebellar tissue types from iPSCs [103–105], and given the increasing interest in using iPSCs to model human neurodevelopment, more differentiation protocols are likely to follow. These will open the door for scientists to interrogate the unusual Na_V_1.2 expression pattern observed in the cerebellum and investigate its contribution to *SCN2A* syndromes.

One particular strength of human iPSC models is that they retain the genetic code of the patients from whom they are derived. Unlike postmortem tissue, iPSC models effectively turn back the clock on a variety of potentially confounding lifetime exposures, such as medication, comorbid diseases, age, and substance use. This yields a powerful tool for determining which genetic pathways may be altered by a specific mutation or set of common variants. In the case of *SCN2A* syndromes, iPSCs from patients who have known deleterious *SCN2A* mutations could help identify which pathways are perturbed during specific developmental stages in specific brain regions, potentially revealing new druggable therapeutic targets and advancing our understanding of this complex syndrome.

### Xenograft studies

Although many human neuronal culture systems are limited in their ability to generate mature neurons with normal electrophysiological properties, xenograft systems have not only met with success in this area, but they have also shown that many of the signaling cues for development and migration of neurons are common between mice and humans. Multiple groups have shown that human iPSC-derived cells introduced into the mouse brain migrate to their proper location and develop mature characteristics of their specified cell type [91, 106, 107]. Although xenograft systems do not mimic a fully human context, they are uniquely well suited for studies of how iPSC-derived neurons integrate into a functional neuronal circuit in a brain, which no current iPSC-only model can (yet) do. As with the organoid studies mentioned above, xenograft studies could allow for more precise analyses of how *SCN2A* mutations affect the assembly and function of neural circuits that rely on *SCN2A*-expressing cells in the cerebral and cerebellar cortices.

## Conclusions

Autism spectrum disorders are a complex group of neuropsychiatric disorders with genetic and environmental causes. A lack of robust cellular models for ASD remains a bottleneck to progress for researchers and clinicians compelled to expand the mechanistic understanding of ASD, as well as those working to develop new therapeutic interventions for ASD management. Monogenic causes of ASD, while rare, hold promise as cellular testbeds for drug development and biological insights alike. While *SCN2A* may not be alone in this potential, the development and critical assessment of *SCN2A* channelopathies in the context of experimental neuronal systems will be an essential step for expanding the palette of experimental systems needed to achieve a better molecular understanding of ASD. *SCN2A* and other as-yet undefined genes may play a role in redefining our understanding of neurodevelopment and ASD.

## Data Availability

Not applicable

## Abbreviations

**5A**: Adult splice isoform of Na_V_1.2
5N: Neonatal splice isoform of Na_V_1.2
ASD: Autism spectrum disorder
CACNA1G: Calcium voltage-gated channel subunit alpha1 G
CaMKII: Calcium/calmodulin dependent protein kinase II
EEG: Electroencephalogram
fMRI: Functional magnetic resonance imaging
hESCs: Human embryonic stem cells
HLF: HLF transcription factor, PAR bZIP family member
ID: Intellectual disability
iPSCs: Induced pluripotent stem cells
KCNQ2: Potassium voltage-gated channel subfamily Q member 2
NEUROG1: Neurogenin 1
NEUROG2: Neurogenin 2
SCN1A: Sodium voltage-gated channel alpha subunit 1
SCN1LAB: Sodium channel, voltage-gated type 1 like, alpha b
SCN2A: Sodium voltage-gated channel alpha subunit 2
SCN3A: Sodium voltage-gated channel alpha subunit 3
